# Effect of the 90-second ‘Gear’ exercise programme on cardiometabolic risk factors in persons with an elevated risk of cardiovascular disease

**DOI:** 10.17159/2078-516X/2021/v33i1a8894

**Published:** 2021-02-09

**Authors:** N Rugbeer, D Constantinou, G Torres

**Affiliations:** 1 Department of Sport, Rehabilitation and Dental Sciences, Tshwane University of Technology, Pretoria, South Africa; 2 Centre for Exercise Science and Sports, School of Therapeutic Sciences, Faculty of Health Sciences University of the Witwatersrand, Johannesburg, South Africa

**Keywords:** lipids, glycaemia, health promotion, cardiovascular health, public health

## Abstract

**Background:**

High-intensity interval training has recently gained popularity at improving cardiometabolic health. However, a close investigation of high-intensity interval training reveals that the exercise duration is similar to moderate-intensity continuous exercise.

**Objective:**

To compare the effect of the time-efficient ‘Gear’ exercise programmes to traditional exercise on cardiometabolic risk factors in persons with an elevated risk of cardiovascular disease.

**Methods:**

The study implemented a six-week, randomised controlled trial. The variables were low-density lipoprotein cholesterol, high-density lipoprotein cholesterol, triglycerides, total cholesterol, glycated haemoglobin (HbA1c), blood pressure and body composition. Forty-eight participants completed the study. Participants were randomly assigned to either the ‘Gear’ exercise programme repeated at different times during the day (GEP-DT): cycled for 90 seconds, repeated three times/day, for three days/week (n = 12); ‘Gear’ exercise programme at one point in time (GEP-OT): cycled for 90 seconds followed by 4 minutes and 30 seconds rest, repeated three times at one point in time, for three days/week (n = 14); 30 minutes of moderate-intensity continuous cycling repeated three days/week at 55–69% HR_max_ (n = 11); and the controls, who were encouraged not to exercise (n = 11).

**Results:**

The 90-second GEP-DT intervention reduced HbA1c post six-weeks of training (MD = 0.1±0.4, % Δ = −1.3%, *d* = −0.70). The GEP-OT group decreased blood triglycerides with a large effect size (MD = 0.6±1.3, % Δ = −31.9%, *d* = −0.83).

**Conclusion:**

The novel 90-second ‘Gear’ exercise programme moderately reduced HbA1c and the 18-minute GEP-OT lowered blood triglycerides. ‘Gear’ exercise programmes will encourage future research in persons with non-communicable diseases, and it should be considered as a public health initiative to promote exercise in clinical, home and work environments.

Cardiovascular disease (CVD) is the leading cause of mortality worldwide. Sedentary behaviour alters metabolic and cardiovascular biomarkers and increases the risk of cardiometabolic diseases. ^[1]^ Exercise is a critical factor to improve cardiometabolic health. Health benefits are associated with following at least 150 minutes of moderate physical activity or 75 minutes of vigorous physical activity per week.^[2]^ Furthermore, the prevention of weight gain is prominent when physical activity is performed at ≥ three metabolic equivalents for >150 minutes per week.^[3]^ However, the optimal duration and intensity of exercise required to improve cardiometabolic health warrants further investigation in the current day and age of limited time and escalating work and family commitments.

High-intensity interval training (HIIT) has recently gained popularity as a time-efficient exercise intervention aimed at improving cardiometabolic health in different population groups. A randomised controlled trial reported that HIIT (9x4 minute intervals at −90% peak oxygen consumption with three minutes rest) was superior at improving insulin sensitivity compared with moderate-intensity continuous exercise (MICE) in overweight women.^[4]^ A contemporary meta-analysis showed that HIIT provided similar benefits to those undertaking MICE for improving body composition and total cholesterol in overweight or obese adults.^[5]^ Thus research seems inconsistent concerning the optimal duration, intensity and type of exercise aimed at improving cardiometabolic health in overweight or obese adults.

A meta-review of 33 systematic reviews reported that HIIT improved some inflammatory markers, cardiac and vascular functions, blood glucose, glycaemic control, cardiorespiratory fitness, heart rate, weight and percentage body fat variables.^[6]^ However, traditional HIIT with repeated sprints may result in undue stress on the cardiovascular system.^[7]^ A close investigation of high-intensity training reveals that it is not a time-efficient intervention considering the repeated high-intensity bouts followed by rest intervals during the exercise session.^[8]^ A brief bout of exercise during different periods of the day will drastically minimise the time commitment of training.^[8]^

Mind-body exercise interventions improve general mental and physical health due to the direct influence of the nervous system on immune and endocrine functions.^[9]^ The ‘Gear’ exercise programme uses visualisation to enable participants to cognitively construct the increase and decrease of gears in a car to represent an increase and decrease in speed and intensity before and during the exercise session. The ‘Gear’ exercise programme is new and this is the first study to test the principles of this exercise programme. Therefore, the current study aimed to investigate the effect of the novel ‘Gear’ exercise programme on cardiometabolic risk factors in persons with an elevated risk of cardiovascular disease.

## Methods

### Study design

The study implemented a randomised controlled pre-test and post-test exercise intervention study design.

### Ethical considerations

The study was conducted in accordance with the Declaration of Helsinki. Ethical clearance was obtained from the University of the Witwatersrand’s Human Research Ethics Committee (Medical) (M170239) and Tshwane University of Technology’s Research Ethics Committee (REC/2016/10/005).

### Population/unit of analysis and sampling strategy

**Fig. 1:**
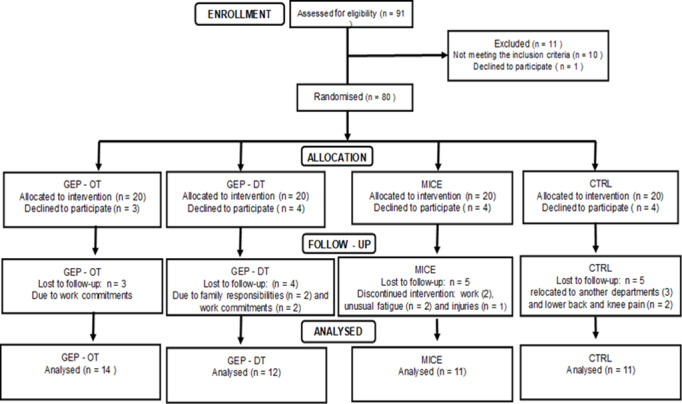
Consort diagram. GEP-OT, ‘Gear’ exercise programme at one point in time; GEP-DT, ‘Gear’ exercise programme repeated at different times during the day; MICE, moderate-intensity continuous exercise; CTRL, control group

Staff from the university were invited to participate in the study voluntarily. Participants were screened using a questionnaire to ensure that they met the inclusion criteria of the study. Participants were included in the study if they were between 30 and 49 years old and had two or more risk factors for CVD, such as physical inactivity, were overweight or obese, elevated blood pressure and elevated lipid profile. The participants should not have had any signs and symptoms of CVD, such as chest pain, dyspnoea, syncope, orthopnoea, tachycardia, known heart murmurs and unusual fatigue with activities of daily living. A physician medically cleared the participants for participation in the study. Using simple random sampling, participants were assigned to the following groups: ‘Gear’ exercise programme repeated at different times during the day (GEP-DT): cycled for 90 seconds, repeated three times/day, for three days/week (n = 12); ‘Gear’ exercise programme at one point in time (GEP-OT): cycled for 90 seconds followed by 4 minutes and 30 seconds rest, repeated three times at one point in time, for three days/week (n = 14); 30 minutes of moderate-intensity continuous cycling repeated three days/week at 55–69% HRmax (n = 11); and the controls, who were encouraged not to exercise (n = 11). Few participants were unable to participate in the follow-up assessments due to work commitments, family responsibilities and orthopaedic injuries not related to the exercise interventions (Fig. 1). Forty-eight participants completed the study (GEP-DT = 14; GEP-OT = 12; MICE = 11; CTRL = 11). The exercise programmes did not result in adverse events (Fig. 1).

#### Lipid profile

A fasting lipid profile was measured using the CardioChek® PA metre (Polymer Technology Systems, Indianapolis, United States of America). The test was conducted after the participants had adhered to an eight-hour overnight fast and was performed according to standard manufacturer procedures. The A1CNow®^+^ (Polymer Technology Systems, Indianapolis, United States of America) was used to measure glycated haemoglobin (HbA1c) using the standard manufacturer recommended procedure.

#### Blood pressure

An appropriately sized blood pressure cuff (Gold Series DS66 Trigger Aneroids, Welch Allyn, United States) was used on the non-dominant arm to measure blood pressure using standardised techniques. Blood pressure was measured twice on the non-dominate arm and the average measurement was documented.

#### Body composition

International Standards for Anthropometric Assessment (ISAK) guidelines were used to measure height (Seca 213, Hamburg, Germany), body mass to the nearest 0.1 kg (Tanita digital scale, model HD-319, Tanita Corporation, Tokyo, Japan), body mass index, skinfolds (Harpenden skinfold calliper, Baty International, West Sussex, United Kingdom), and waist and hip circumferences.

#### Exercise programmes

Blood pressure and heart rate were documented before the commencement of the exercise session. The exercise session was conducted three times/week for six weeks on alternate days of the week. The mode of exercise was stationary cycling on a wattbike with the air resistance set at 1 to facilitate cycling speed (Wattbike Pro, Woodway, Germany). Dietary and fluid intake were not controlled. The researcher (biokineticist) supervised all exercise sessions. The new proposed GEP-DT and GEP-OT integrates and stimulates the function of the mind and body during the exercise session using visualisation. The GEP-DT commenced with a visualisation process. A car analogy was used to explain the exercise intervention. The participants were encouraged to visualise the increase and decrease of gears in a car to represent the increase and decrease of speed and intensity in the exercise intervention respectively. The speed and intensity at the different gears were mentally constructed. The gears remained constant on the bike. The participants increased the gears mindfully, relevant to their perceived increase in speed and intensity, which was self-paced, enabling them to be in control of the exercise session.

**Fig. 2:**
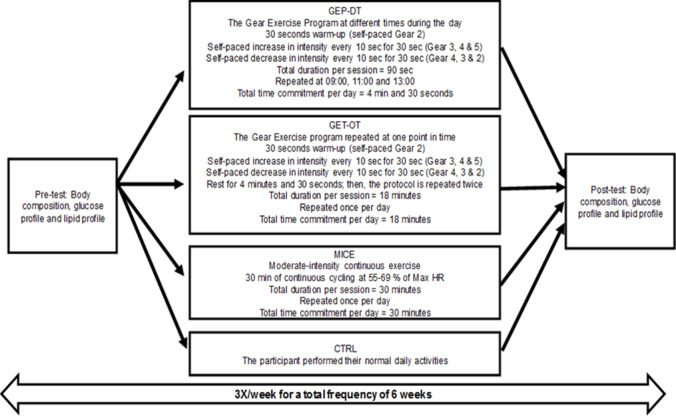
Exercise programmes. GEP-OT, ‘Gear’ exercise programme at one point in time; GEP-DT, ‘Gear’ exercise programme repeated at different times during the day; MICE, moderate-intensity continuous exercise; CTRL, control group

Once the visualisation process was completed, the participants commenced the GEP-DT with a 30 second warmup (self-paced visualisation gear 2). Participants then increased the intensity gradually over 30 seconds using the visualisation technique (visualisation gear 3 = 10 seconds, visualisation gear 4 = 10 seconds and visualisation gear 5 = 10 seconds). After reaching peak self-paced speed at visualisation gear 5, the participants reduced the speed by decreasing the intensity every 10 seconds for 30 seconds (visualisation gear 4 = 10 seconds, visualisation gear 3 = 10 seconds and visualisation gear 2 = 10 seconds). Hence, participants geared up and down in a self-controlled manner, which acted as a stimulus for them to be mindful of the exercise session. The total duration of the GEP-DT per session was 90 seconds. This exercise intervention was repeated three times a day, with a total time commitment of 4 minutes and 30 seconds per day (Fig. 2). The GEP-OT was similar to the GEP-DT protocol; however, participants repeated three repetitions of the intervention at one point in time with a rest period of 4 minutes and 30 seconds per repetition. Therefore, the total duration of the GEP-OT was 18 minutes per session (Fig. 2). Participants in the MICE group performed moderate-intensity continuous cycling for a total duration of 30 minutes at 55–69% of HR_max_. The participants in the control group (CTRL) did not exercise and continued with their daily administrative and household tasks, which were low-intensity activities (<3 metabolic equivalents) (Fig. 2). A demonstration of the ‘Gear’ exercise programme can be viewed on the following link https://www.youtube.com/watch?v=KAbkRg9ex94

### Statistical analysis

Data were analysed using SPSS Statistics for Windows, version 23.0 (SPSS Inc., Chicago, Ill., USA). The study was powered to detect between-group differences using G*Power version 3 (≥10 participants per group with an acquired physiological effect size of 0.25 at *α* = 0.05). Normality of data was analysed using the Shapiro-Wilk test, histograms and Q-Q plots. Non-normal variables were log-transformed and thereafter the variables were included in the analysis. Univariate analysis was conducted to determine baseline differences in age, fitness level and body weight between the subject groups. A paired samples t-test was used to determine intra-group differences in the exercise interventions. The repeated measures analysis of variance (ANOVA) was used to compare intergroup differences. If interaction effects were observed, the Bonferroni post hoc test was used to compare treatment effects between the various groups. Effect size (*d)* was calculated to determine the clinical significance between the exercise groups and the control group. The effect size was classified according to small effect size (*d* ≥ 0.20), moderate effect size (*d* ≥ 0.5) and large effect size (d ≥ 0.8), respectively. The effect size was accepted as a true effect if the effect size confidence intervals did not cross both the positive and negative 0.20 thresholds. The effect size was considered to be unclear if this occurred. The level of significance was set at p<0.05.

## Results

The mean age of participants in the training groups was GEP-OT = 37 years, GEP-DT = 39 years, MICE = 40 years and CTRL = 38 years. More women than men participated in the study. There was no significant difference of age, fitness level and body mass between the subject groups at baseline (Table 1). The GEP-DT group had the highest adherence rate (87%) and the shortest significant exercise time commitment of 4 minutes and 30 seconds compared with the GEP-OT (18 minutes) and the MICE group (30 minutes). There was a significant difference in kilocalories in all the exercise groups. The average kilocalorie expenditure in the MICE group was 16 times that of the GEP-DT. There was a significant difference in peak heart rate, average power, peak power, average power/body mass, peak power/body mass and cadence between the GEP-DT vs MICE and the GEP-OT vs MICE. The GEP-OT (129±13 bpm) had the highest peak exercise heart rate compared to MICE and GEP-DT groups. Moreover, the GEP-DT (110±9 bpm) had the highest average exercise heart rate compared to the other groups (Table 2).

There was a significant decrease in the mean diastolic blood pressure within the MICE group (p = 0.01). The systolic blood pressure and body composition variables were insignificant with the subject groups (p>0.05) (Table 3). There was a significant increase in glycated haemoglobin within the MICE group (p = 0.01). The other metabolic variables showed insignificant differences within the subject groups (p>0.05) (Table 4).

The MICE group demonstrated a large effect size for diastolic blood pressure when compared to the CTRL group, which showed a true large meaningful reduction in diastolic blood pressure post-training (MD = −5.4±3.8, % Δ = −6.8%, *d* = −0.81, *d* (95% CI): −1.68, 0.06). This group demonstrated a large effect size for percentage body fat compared to the CTRL group, which showed a true large meaningful reduction in percentage body fat post-training (MD = 0.7±2.1, % Δ = −2.5, *d* = −0.83, *d* (95% CI): −1.70, 0.04) (Table 5). Although the MICE group showed a moderate effect size for body mass and body mass index, the confidence intervals crossed the lower positive threshold (0.20); therefore, the effect is unclear for body mass and body mass index in the MICE group (Table 5). Furthermore, the effect is unclear for percentage body fat and body mass index in the GEP-DT group (Table 5).

The 90-second GEP-DT intervention reduced glycated haemoglobin post six weeks of training with a moderate effect size compared to the CTRL group, which showed a true moderate meaningful reduction in glycated haemoglobin post-training (MD = −0.1±0.4, % Δ = −1.3%, *d* = −0.70, d (95% CI): −1.55, 0.14) (Table 6). This group demonstrated a large effect size for low-density lipoprotein cholesterol/high-density lipoprotein cholesterol, which showed a true large meaningful increase in low-density lipoprotein cholesterol/high-density lipoprotein cholesterol post-training (MD = 0.2±0.6, % Δ = 8.2, *d* = 0.89, d (95% CI): −0.05, 1.84) (Table 6). The GEP-OT group demonstrated a decrease in triglycerides post-training with a large effect size when compared to the CTRL group, which showed a true large meaningful reduction in triglycerides post-training (MD = 0.6±1.3, % Δ = −31.9%, *d* = −0.83, d (95% CI): −1.68, 0.01) (Table 6). Although the MICE group showed a moderate effect size for triglycerides, the confidence intervals crossed the lower positive threshold (0.20); therefore, the effect is unclear for triglycerides in the MICE group (Table 6).

Analysis of covariance showed no significant group x time interactions on blood pressure, body composition and metabolic markers post six weeks of exercise in persons with an elevated risk of cardiovascular disease (p>0.05) (Tables 5 and 6).

## Discussion

The primary finding of the study was that the 90-second GEP-DT intervention moderately decreased HbA1c in comparison to the CTRL group. The participants in the GEP-DT significantly expended fewer calories compared to the other exercise groups, which suggests that the benefits derived from the GEP-DT intervention are less reliant on calories expended during the exercise session. There was no significant difference in age, fitness level and body mass between the groups at baseline, which did not influence the effect of the exercise interventions. Glycated haemoglobin is the gold standard to measure glycaemic control,^[10]^ and an absolute change of 1% reduction in HbA1c is associated with 37% reduction in microvascular complications, 21% reduction in diabetes-related mortality and 14% of myocardial infarction.^[11]^ Furthermore, an absolute decrease of ≥0.3% in HbA1c is considered a clinically meaningful value by the European medicine agencies.^[12]^ The study showed a clinically significant moderate effect at a comparatively lower absolute change of 0.1%. However, this was achieved at a lower exercise volume compared to traditional models of training. Diet could have influenced the HbA1c result because the study did not control the carbohydrate intake.

A systematic review and meta-analysis investigating the effects of familiar MICE and HIIT protocols revealed no significant differences in lipid profiles between the groups.^[13]^ The current study concurs with the finding of this review; however, the novel finding is that the new GEP-OT lowered triglyceride levels in persons with an elevated risk of cardiovascular disease with a large effect size irrespective of the insignificant probability value between the respective groups due to the small sample size. Non-high-density lipoprotein cholesterol and elevated triglycerides are crucial mediators for residual atherosclerotic cardiovascular disease risk.^[14]^ Thus, the GEP-OT group demonstrated a large decrease in effect size for triglycerides post six weeks of training, which provides an atheroprotective and cardioprotective effect in persons with an elevated risk of cardiovascular disease. Although the 90-second GEP-DT raised the low-density lipoprotein cholesterol/high-density lipoprotein cholesterol, a study indicated that enhancing the function rather than increasing the levels of high-density lipoprotein cholesterol is associated with a clinical effect in lowering the risk of CVD.^[15]^

Moderate-intensity continuous exercise significantly decreased diastolic blood pressure (DBP). An international Korean longitudinal study revealed a J-shaped relationship relevant to DBP in the general population.^[16]^ The Korean study suggested that the general population is at risk of all-cause mortality with a DBP of <60 mmHg.^[16]^ The reason for this finding is that coronary perfusion occurs during diastole. Coronary blood flow to the myocardium is hindered when DBP is <60 mmHg. Moderate-intensity continuous exercise decreased DBP in the current study; however, the participants’ mean DBP post-exercise was >60 mmHg, which had a cardioprotective effect in this group of participants. MICE reduced % body fat with a large effect size. This finding concurs with a pilot randomised controlled trial showing a reduced body fat and body weight after engaging in light-intensity training. ^[17]^ Moderate-intensity continuous exercise releases non-esterified fatty acids and oxidises free fatty acids. This increases the capacity of the muscle to take up non-esterified fatty acids by enhancing the requirement and number of capillaries, which stimulates the mobilisation of abdominal adipose tissue non-esterified fatty acids and decreases the risk of cardiovascular disease. ^[18]^

### Limitations

More women than men participated in this study, and the study did not account for the potential effect of the different phases of the menstrual cycle. The study also did not control nutrition status. The control group’s activities were self-reported and not objectively measured. Moreover, the study had a small sample size because it was powered from traditional high-intensity programmes.

## Conclusion

The novel 90-second ‘Gear’ exercise programme moderately reduced HbA1c and the 18-minute GEP-OT lowered blood triglycerides. Moderate-intensity continuous exercise decreased diastolic blood pressure and % body fat. The ‘Gear’ exercise programmes will encourage future research in persons with non-communicable diseases and should be considered as a public health initiative to promote exercise in clinical, home and work environments.

## Figures and Tables

**Table 1:** Demographic profile of participants

Variable	GEP-OT (n=14)	GEP-DT (n=12)	MICE (n=11)	CTRL (n=11)	p-value
The ratio of women to men	11/3	12/0	8/3	9/2	N/A
Age (years)	37 ± 5	39 ± 7	40 ± 6	38 ± 5	0.68
Body mass (kg)	79.9 ± 11.9	85.1 ± 23.4	83.2 ± 14.7	84.6 ± 17.9	0.87
VO_2peak_ (mL·min^−1^·kg^−1^)	11.7 ± 3.8	11.7 ± 3.5	12.3 ± 4.1	10.1 ± 3.3	0.56

Data are presented as mean ± standard deviation. n, number; N/A, not applicable; GEP-OT, ‘Gear’ exercise programme at one point in time; GEP-DT, ‘Gear’ exercise programme at different times during the day; MICE, moderate-intensity continuous exercise; CTRL, control group; VO_2peak_, peak oxygen consumption; p-value, probability value

**Table 2:** Training data of participants in the exercise intervention groups

Training variables	GEP-OT (n=14)	GEP-DT (n=12)	MICE (n=11)	p-value
Adherence (%)	82	87	77	N/A
Time commitment (minutes)	18	4.5	30	0.001*#&
Average heart rate (bpm)	109 ± 10	110 ± 9	109 ± 5	0.24
Average heart rate expressed as % of max heart rate	60 ± 5	61 ± 4	61 ± 3	0.35
Peak heart rate (bpm)	129 ± 13	127 ± 12	120 ± 6	0.001*#
Peak heart rate expressed as % of max heart rate	71 ± 7	70 ± 6	67 ± 3	0.001*#
Average power (W)	62 ± 33	58 ± 23	31 ± 15	0.001*#
Peak power (W)	127 ± 68	117 ± 56	52 ± 28	0.001*#
Average power/body mass (W·kg^−1^)	0.76 ± 0.37	0.76 ± 0.55	0.38 ± 0.20	0.001*#
Peak power/body mass (W·kg^−1^)	1.58 ± 0.80	1.54 ± 0.88	0.62 ± 0.29	0.001*#
Kilocalories (Kcal)	66.2 ± 9.5	9.3 ± 2.1	148.0 ± 27.5	0.001*#&
Cadence (RPM)	57 ± 13	54 ± 8	44 ± 10	0.001*#
Peak cadence (RPM)	75 ± 17	73 ± 8	52 ± 11	0.001*#

Data are presented as mean ± standard deviation. %, percentage; bpm, beats per minute; W, watts; W·kg·1,watts/kilograms; Kcal, kilocalories; RPM, revolutions per minute; GEP-OT, ‘Gear’ exercise programme at one point in time; GEP-DT, ‘Gear’ exercise programme repeated at different times during the day; MICE, moderate-intensity continuous exercise; NA, not applicable; p-value, probability value.

* Significant difference between MICE vs GEP-DT (p<0.01).

# Significant difference between MICE vs GEP-OT (p<0.01).

& Significant difference between GEP-OT vs GEP-DT (p<0.01)

**Table 3:** Intra-group comparisons of blood pressure and body composition measurements across the subject groups

Variables	GEP-OT (n=14)	GEP-DT (n=12)	MICE (n=11)	CTRL (n=11)
**Systolic BP (mmHg)**				
Pre-test	113.0 ± 13.1	111.0 ± 13.6	113.0 ± 12.0	110.0 ± 13.0
Post-test	110.9 ± 11.5	108.0 ± 8.30	108.7 ± 12.3	105.2 ± 10.5
p-value	0.38	0.37	0.16	0.07

**Diastolic BP (mmHg)**				
Pre-test	79.1 ± 5.98	78.4 ± 7.33	80.0 ± 9.48	78.73 ± 6.50
Post-test	77.3 ± 7.91	78.0 ± 6.38	74.6 ± 9.59	77.36 ± 6.86
p-value	0.34	0.84	0.00^a^	0.46

**Body mass (Kg)**				
Pre-test	79.9 ± 12.0	85.1 ± 23.4	83.2 ± 14.7	84.6 ± 18.0
Post-test	80.2 ± 11.6	85.6 ± 23.8	83.2 ± 14.2	85.5 ± 18.4
p-value	0.68	0.31	1.00	0.20

**BMI (Kg/m** **^2^** **)**				
Pre-test	29.5 ± 4.71	32.2 ± 9.80	31.0 ± 5.44	31.3 ± 5.09
Post-test	29.8 ± 4.86	32.2 ± 9.59	31.0 ± 5.51	31.8 ± 5.52
p-value	0.31	0.93	0.99	0.08

**Waist circumference (cm)**				
Pre-test	90.6 ± 8.97	89.8 ± 19.1	93.7 ± 11.8	97.1 ± 12.8
Post-test	88.4 ± 9.39	91.3 ± 14.4	91.5 ± 10.4	95.3 ± 10.9
p value	0.12	0.57	0.28	0.23

**Body fat (%)**				
Pre-test	27.3 ± 7.10	28.9 ± 6.21	27.9 ± 6.41	28.0 ± 4.93
Post-test	27.7 ± 6.37	28.7 ± 5.48	27.2 ± 5.57	29.3 ± 6.65
p-value	0.65	0.78	0.28	0.14

Data are presented as mean ± standard deviation.

a , Intra-group significance p<0.05;

GEP-OT, ‘Gear’ exercise programme at one point in time; GEP-DT, ‘Gear’ exercise programme at different times during the day; MICE, moderate-intensity continuous exercise; CTRL, control group; BP, blood pressure; BMI, body mass index

**Table 4:** Intra-group comparisons of metabolic markers across the subject groups

Variables	GEP-OT	GEP-DT	MICE	CRTL
**Total cholesterol (mmol/L)**				
N	14	12	11	11
Pre-test	3.91 ± 0.91	4.17 ± 0.89	4.46 ± 1.08	4.23 ± 1.00
Post-test	3.86 ± 0.75	4.12 ± 1.20	4.33 ± 0.94	4.32 ± 1.04
p-value	0.88	0.78	0.43	0.76

**HDL-C (mmol/L)**				
N	14	12	11	11
Pre-test	1.24 ± 0.35	1.26 ± 0.27	1.40 ± 0.37	1.15 ± 0.21
Post-test	1.38 ± 0.36	1.27 ± 0.31	1.48 ± 0.49	1.23 ± 0.24
p-value	0.08	0.74	0.24	0.15

**LDL-C (mmol/L)**				
N	11	9	9	10
Pre-test	2.03 ± 0.84	2.15 ± 0.0.55	2.31 ± 0.82	2.58 ± 0.82
Post-test	1.91 ± 0.77	2.38 ± 1.10	2.13 ± 0.66	2.45 ± 0.85
p-value	0.69	0.42	0.41	0.60

**Triglycerides (mmol/L)**				
N	14	12	9	10
Pre-test	1.85 ± 1.38	1.84 ± 0.89	1.24 ± 0.59	1.37 ± 0.48
Post-test	1.26 ± 0.49	2.04 ± 1.39	1.23 ± 0.49	1.72 ± 0.85
p-value	0.15	0.70	0.98	0.19

**TC/HDL-C**				
N	14	12	11	11
Pre-test	3.37 ± 1.23	3.39 ± 0.84	3.32 ± 0.91	3.81 ± 1.12
Post-test	2.94 ± 0.79	3.32 ± 0.96	3.13 ± 0.94	3.58 ± 1.05
p-value	0.10	0.46	0.20	0.10

**LDL-C/HDL-C**				
N	11	9	9	10
Pre-test	1.82 ± 0.97	1.84 ± 0.55	1.86 ± 0.79	2.36 ± 0.88
Post-test	1.57 ± 0.74	1.99 ± 0.83	1.68 ± 0.86	2.02 ± 0.81
p-value	0.43	0.49	0.36	0.06

**Non-HDL-C (mmol/L)**				
N	14	12	11	11
Pre-test	2.67 ± 0.91	2.90 ± 0.81	3.06 ± 1.02	3.09 ± 0.99
Post-test	2.48 ± 0.73	2.85 ± 1.10	2.85 ± 0.87	3.02 ± 1.06
p-value	0.47	0.75	0.15	0.82

**Fasting glucose (mmol/L)**				
N	14	12	11	11
Pre-test	5.07 ± 0.60	4.86 ± 0.60	5.16 ± 0.76	5.02 ± 0.43
Post-test	4.92 ± 0.48	4.89 ± 0.54	5.28 ± 0.46	5.05 ± 0.34
p-value	0.27	0.84	0.55	0.76

**HbA1C (%)**				
N	14	12	11	11
Pre-test	5.90 ± 0.40	6.02 ± 0.34	5.78 ± 0.28	5.93 ± 0.31
Post-test	6.01 ± 0.46	5.94 ± 0.36	5.97 ± 0.26	6.10 ± 0.44
p-value	0.26	0.49	0.00^a^	0.12

Data are presented as mean ± standard deviation.

a , Intra-group significance p<0.05;

N, number of participants; GEP-OT, ‘Gear’ exercise programme at one point in time; GEP-DT, ‘Gear’ exercise programme at different times during the day; MICE, moderate-intensity continuous exercise; CTRL, control group; HDL-C, high-density lipoprotein cholesterol; LDL-C, low-density lipoprotein cholesterol; TC/HDL-C, total cholesterol/high-density lipoprotein cholesterol; LDL-C/HDL-C, low-density lipoprotein cholesterol/high-density lipoprotein cholesterol; Non-HDL-C, non-high-density lipoprotein cholesterol; HbA1C, glycated haemoglobin

**Table 5:** Inter-group comparisons of blood pressure and body composition measurements across the subject groups

Variables	GEP-OT	GEP-DT	MICE	CRTL	p-value (GxT)
**Systolic BP (mmHg)**					
Δ Score	−2.1 ± 8.9	−3.0 ± 11.2	−4.3 ± 9.4	−4.8 ± 8.0	0.89
% Δ	−1.9	−2.7	−3.8	−4.4	
*d* (95% C.I.)	0.32 (−0.48, 1.11)	0.18 (−0.64, 1.00)	0.06 (−0.78, 0.89)	N/A	

**Diastolic BP (mmHg)**					
Δ Score	−1.8 ± 6.7	−0.4 ± 6.9	−5.4 ± 3.8	−1.4 ± 5.9	0.24
% Δ	−2.3	−0.5	−6.8	−1.7	
*d* (95% C.I.)	−0.07 (−0.86, 0.72)	0.15 (−0.67, 0.97)	−0.81^c^ (−1.68, 0.06)	N/A	

**Body mass (Kg)**					
Δ Score	0.3 ± 2.5	0.5 ± 1.7	0.0 ± 1.5	0.9 ± 2.0	0.86
% Δ	0.4	0.6	0.0	1.1	
*d* (95% C.I.)	−0.26 (−1.05, 0.53)	−0.22 (−1.04, 0.60)	−0.51 (−1.36, 0.34)	N/A	

**BMI (Kg/m** **^2^** **)**					
Δ Score	0.3 ± 1.1	0.0 ± 0.9	0.0 ± 0.6	0.5 ± 0.9	0.49
% Δ	1.0	0.0	0.0	1.6	
*d* (95% C.I.)	−0.20 (−0.99, 0.60)	0.56 (−1.39, 0.28)	−0.65 (−1.51, 0.20)	N/A	

**Waist circumference (cm)**					
Δ Score	−2.2 ± 5.1	1.5 ± 8.4	−2.2 ± 6.6	−1.8 ± 4.7	0.42
% Δ	−2.4	1.7	−2.4	−1.9	
*d* (95% C.I.)	−0.08 (−0.87, 0.71)	0.48 (−0.35, 1.31)	−0.07 (−0.91, 0.77)	N/A	

**Body fat (%)**					
Δ Score	0.4 ± 2.7	−0.2 ± 2.8	−0.7 ± 2.1	1.3 ± 2.7	0.31
% Δ	1.5	−0.7	−2.5	4.6	
*d* (95% C.I.)	−0.33 (−1.13, 0.46)	−0.55 (−1.38, 0.29)	−0.83 c (−1.70, 0.04)	N/A	

Δ Score, mean difference ± standard deviation of mean difference (absolute change score); % Δ, relative percent change from baseline (relative change score); p-value (GxT), the interaction probability value between the subject groups and time from the repeated measures analysis of variance; d (95% C.I.), effect size (95% confidence interval of effect size);

c , large effect size (compared to the control group);

N/A, not applicable; GEP-OT, ‘Gear’ exercise programme at one point in time; GEP-DT, ‘Gear’ exercise programme at different times during the day; MICE, moderate-intensity continuous exercise; CTRL, control group; BP, blood pressure; BMI, body mass index

**Table 6:** Inter-group comparisons of metabolic markers across the subject groups

Variables	GEP-OT	GEP-DT	MICE	CRTL	p-value (TxG)
**Total cholesterol (mmol/L)**					
Δ Score	−0.1 ± 1.0	−0.1 ± 0.6	−0.1 ± 0.5	0.1 ± 1.0	0.94
% Δ	−1.3	−1.2	−2.9	2.1	
d (95% C.I.)	−0.14 (−0.93, 0.65)	−0.17 (−0.99, 0.65)	−0.28 (−1.12, 0.56)	N/A	

**HDL-C (mmol/L)**					
Δ Score	0.1 ± 0.3	0.0 ± 0.1	0.1 ± 0.2	0.1 ± 0.2	0.28
% Δ	11.	0.8	5.7	7.0	
d (95% C.I.)	0.23 (−0.56, 1.02)	−0.43 (−1.26, 0.40)	0.00 (−0.84, 0.84)	N/A	

**LDL-C (mmol/L)**					
Δ Score	−0.1 ± 1.1	0.2 ± 0.8	−0.2 ± 0.6	−0.1 ± 0.8	0.69
% Δ	−5.9	10.7	−7.8	−5.0	
d (95% C.I.)	0.01 (−0.85, 0.87)	0.45 (−0.46, 1.36)	−0.07 (−0.97, 0.83)	N/A	

**Triglycerides (mmol/L)**					
Δ Score	−0.6 ± 1.3	0.2 ± 1.6	−0.0 ± 0.6	0.4 ± 0.8	0.44
% Δ	−31.9	10.9	−0.8	25.5	
d (95% C.I.)	−0.83^c^ (−1.68, 0.01)	−0.11 (−0.95, 0.73)	−0.50 (−1.42, 0.41)	N/A	

**TC/HDLC**					
Δ Score	−0.4 ± 0.9	−0.1 ± 0.3	−0.2 ± 0.5	−0.2 ± 0.4	0.63
% Δ	−12.8	−2.1	−5.7	−6.04	
d (95% C.I.)	−0.28 (−1.07, 0.52)	0.43 (−0.40, 1.26)	0.09 (−0.75, 0.92)	N/A	

**LDL-C/HDL-C**					
Δ Score	−0.3 ± 1.0	0.2 ± 0.6	−0.2 ± 0.5	−0.3 ± 0.5	0.73
% Δ	−13.7	8.2	−9.7	−14.4	
d (95% C.I.)	0.11 (−0.74, 0.97)	0.89 c (−0.05, 1.84)	0.32 (−0.59, 1.23)	N/A	

**Non-HDL-C (mmol/L)**					
Δ Score	−0.2 ± 0.9	−0.1 ± 0.6	−0.2 ± 0.5	−0.1 ± 0.9	0.99
% Δ	−7.1	−1.7	−6.9	−2.3	
d (95% C.I.)	−0.13 (−0.92, 0.66)	0.03 (−0.79, 0.85)	−0.19 (−1.03, 0.65)	N/A	

**Fasting glucose (mmol/L)**					
Δ Score	−0.2 ± 0.5	0.0 ± 0.4	0.1 ± 0.7	0.0 ± 0.3	0.55
% Δ	−3.	0.6	2.3	0.6	
d (95% C.I.)	−0.42 (−1.22, 0.37)	0.00 (−0.82, 0.82)	0.18 (−0.66, 1.01)	N/A	

**HbA1C (%)**					
Δ Score	0.1 ± 0.4	−0.1 ± 0.4	0.2 ± 0.1	0.2 ± 0.3	0.18
% Δ	1.9	−1.3	3.3	2.9	
*d* (95% C.I.)	−0.17 (−0.96, 0.62)	−0.70^b^ (−1.55, 0.14)	0.09 (−0.75, 0.93)	N/A	

Δ Score, mean difference ± standard deviation of mean difference (absolute change score); % Δ, relative percent change from baseline (relative change score); p-value (GxT), the interaction probability value between the subject groups and time from the repeated measures analysis of variance; d (95% C.I.), effect size (95% confidence interval of effect size);

b , moderate effect size (compared to the control group);

c , large effect size (compared to the control group);

N/A, not applicable; GEP-OT, ‘Gear’ exercise programme at one point in time; GEP-DT, ‘Gear’ exercise programme at different times during the day; MICE, moderate-intensity continuous exercise; CTRL, control group; HDL-C, high-density lipoprotein cholesterol; LDL-C, low-density lipoprotein cholesterol; TC/HDL-C, total cholesterol/high-density lipoprotein cholesterol; LDL-C/HDL-C, low-density lipoprotein cholesterol/high-density lipoprotein cholesterol; Non-HDL-C, non-high-density lipoprotein cholesterol; HbA1C, glycated haemoglobin
